# Sequence-Signature Optimization Enables Improved Identification of Human HV6-1-Derived Class Antibodies That Neutralize Diverse Influenza A Viruses

**DOI:** 10.3389/fimmu.2021.662909

**Published:** 2021-05-31

**Authors:** Gwo-Yu Chuang, Chen-Hsiang Shen, Crystal Sao-Fong Cheung, Jason Gorman, Adrian Creanga, M. Gordon Joyce, Kwanyee Leung, Reda Rawi, Lingshu Wang, Eun Sung Yang, Yongping Yang, Baoshan Zhang, Yi Zhang, Masaru Kanekiyo, Tongqing Zhou, Brandon J. DeKosky, Barney S. Graham, John R. Mascola, Peter D. Kwong

**Affiliations:** ^1^ Vaccine Research Center, National Institute of Allergy and Infectious Diseases, National Institutes of Health, Bethesda, MD, United States; ^2^ Department of Pharmaceutical Chemistry and Department of Chemical Engineering, University of Kansas, Lawrence, KS, United States

**Keywords:** antibody identification, hemagglutinin stem, influenza, iterative optimization, multidonor class antibody, neutralizing antibody, sequence signature

## Abstract

Sequence signatures of multidonor broadly neutralizing influenza antibodies can be used to quantify the prevalence of B cells with virus-neutralizing potential to accelerate development of broadly protective vaccine strategies. Antibodies of the same class share similar recognition modes and developmental pathways, and several antibody classes have been identified that neutralize diverse group 1- and group 2-influenza A viruses and have been observed in multiple human donors. One such multidonor antibody class, the HV6-1-derived class, targets the stem region of hemagglutinin with extraordinary neutralization breadth. Here, we use an iterative process to combine informatics, biochemical, and structural analyses to delineate an improved sequence signature for HV6-1-class antibodies. Based on sequence and structure analyses of known HV6-1 class antibodies, we derived a more inclusive signature (version 1), which we used to search for matching B-cell transcripts from published next-generation sequencing datasets of influenza vaccination studies. We expressed selected antibodies, evaluated their function, and identified amino acid-level requirements from which to refine the sequence signature (version 2). The cryo-electron microscopy structure for one of the signature-identified antibodies in complex with hemagglutinin confirmed motif recognition to be similar to known HV6-1-class members, MEDI8852 and 56.a.09, despite differences in recognition-loop length. Threading indicated the refined signature to have increased accuracy, and signature-identified heavy chains, when paired with the light chain of MEDI8852, showed neutralization comparable to the most potent members of the class. Incorporating sequences of additional class members thus enables an improved sequence signature for HV6-1-class antibodies, which can identify class members with increased accuracy.

## Introduction

Understanding the elicitation of broadly neutralizing antibodies (bNAbs) is key to the development of B cell-based vaccines against pathogens of high sequence diversity such as HIV-1 and influenza A virus (1, 2). While B cells from different individuals generally develop unique antibodies against the same antigen, multidonor class antibodies – antibodies from different individuals having similar developmental pathways and targeting antigens with similar modes of recognition – have been identified from sera of donors infected by or vaccinated against pathogens such as HIV-1 (3–8), influenza A virus (9–16), Ebola virus (17–19), dengue virus (20), SARS-CoV-2 (21–32) or malaria parasites (33, 34). Because of the potential reproducibility of these antibodies in the general population, multidonor class antibodies have become prime templates for B cell-based vaccines (35–40).

Sequence signatures have been developed to facilitate the identification of antibodies of the same class (6, 7, 9, 17, 41–43), as such signatures in combination with longitudinal sequencing of B-cell transcripts can be used to monitor the development of antibody class lineages during infection or vaccination studies. For example, sequence signature for VRC01-class antibodies (3, 41), whose members comprise some of the most effective neutralizers of HIV-1 (4, 44–46), have been used to define lineages in humans (7, 47–49) and to monitor the development of HIV-1 neutralizing antibodies both in animal models (50–54) and in a clinical trial (NCT03547245). Likewise, the sequence signature for HV1-69 influenza antibodies targeting the stem region of the hemagglutinin has been characterized and demonstrated also to be allele-specific (42). Sequence signatures have also been identified for mAb114, a therapeutic antibody for the treatment of Ebola virus disease undergoing clinical trial (55), based on antibodies elicited from vaccination of rhesus macaques with Zaire ebolavirus glycoprotein (17).

Another example of a multidonor antibody class is the HV6-1-derived influenza antibodies that target the stem region of influenza hemagglutinin with extraordinary breadth (9, 31, 56). These antibodies utilize heavy chain HV6-1 and HD3-3 genetic elements, and a first-generation antibody-class sequence signature could identify other class members from sequence databases (9). However, MEDI8852 (56), a HV6-1 influenza antibody identified at the same time in a separate study, did not satisfy the first-generation signature, despite clearly having a similar mode of recognition and being of the same class. Specifically, the CDR-H3 length and recognition motif of MEDI8852 were different from what have been delineated by the first-generation signature, indicating the first-generation antibody class-sequence signature was not inclusive enough. In this study we developed a workflow, coupling antigenic screening and bioinformatics analyses to optimize iteratively the HV6-1-class sequence signature. We searched for matching B-cell transcripts, expressed identified antibodies, evaluated their function, and identified residues from which to refine the sequence signature. We assessed signature-identified antibodies and determined the cryo-electron microscopy (cryo-EM) structure of one in complex with hemagglutinin. Overall, we improved the accuracy of the sequence signature for HV6-1-class influenza antibodies and demonstrated that sequence transcripts identified by the optimized sequence-signature are functional, with several that have neutralization activity on par with the best known HV6-1 class antibodies.

## Methods

### Identification of HV6-1 Class Heavy Chain Transcripts Based on Signature Search

Publicly available deep sequencing datasets associated with influenza vaccine trials and heavy chain sequences of healthy donors were downloaded using accession numbers listed in supplemental **Tables S1A, B**. The Stand‐alone IgBLAST (57) was used for V(D)J germline gene assignments, and IgBLAST output was parsed and analyzed by in‐house developed python script. For 454 sequencing, non‐immunoglobin reads and non‐productive reads were removed. For Illumina sequencing, non‐Ig reads, non‐duplicate and non‐productive reads were filtered out. The remaining reads were sieved by the HV6-1 class signatures previously published (9) or developed in this paper. The CDR-H3 of HV6-1 class signature positive reads were clustered with 97 percent sequence identity using CD-HIT (58). The centroid sequence was selected as representative sequence of each cluster for neutralization assay. The germline amino acids were used to repair missing N- and C- terminal residues.

### Microplate-Based Antigenic Analysis

24 h prior to DNA-transient transfection, 100 μl per well of log-phase growing HEK 293T cells were seeded into a 96-well microplate at a density of 2.5x10^5^ cells/ml in optimized expression medium (RealFect-Medium, ABI Scientific, VA), and incubated at 37°C, 5% CO_2_ for 24 hours. Prior to transfection, 40 μl per well of spent medium was removed. For transient transfection, 0.15 ug of each heavy chain variant plasmid DNA was paired with 0.15 ug of light chain plasmid DNA of 56.a.09, 54.f.01, or MEDI8852, respectively, in 10 μl of Opti-MEM medium (Invitrogen, CA) per well in a 96-well plate, and then mixed with 0.9 μl per well of TrueFect-Max transfection reagent (United BioSystems, VA) in 10 μl of Opti-MEM medium, followed by an incubation for 15 min at room temperature (RT). The DNA-TrueFect-Max complex was mixed with growing cells in the 96-well microplate and incubated at 37°C, 5% CO_2_. In day one post transfection, 30 μl per well of enriched expression medium (CelBooster Cell Growth Enhancer Medium for Adherent Cell, ABI Scientific, VA) was fed. After five days post transfection, the antigenic analysis of paired antibodies was characterized by 96-well-formatted ELISA. Briefly, 100 ul per well of Flu hemagglutinin of A/California/04/2009 (CA2009, H1 subtype) or H3 A/Hong Kong/1/1968 (HK1968, H3 subtype), respectively, at a concentration of 4 ug/ml in phosphate buffered saline (PBS) was captured in Ni-coated 96-well ELISA plates (Thermo, IL)) and incubated for two hours at room temperature, followed by the removal of the Flu HA antigen solution and incubation of 200 μl per well of CelBooster Cell Growth Enhancer Medium for Adherent Cell for one hour at RT. After washing with PBS + 0.05% Tween 20, 30 μl per well of the expressed supernatant antibody mixed with 70 μl of PBS was incubated for one hour at RT. After washing, 100 μl per well of Horseradish peroxidase (HRP)-conjugated goat anti-human IgG antibody (Jackson ImmunoResearch Laboratories Inc., PA), diluted at 1:10,000 in CelBooster Cell Growth Enhancer Medium for Adherent Cell with 0.02% tween 20, was incubated for 30 min at RT. After washing, the reaction signal was developed using 100 μl of BioFX-TMB (SurModics, MN) at RT for 10 min, and then stopped with 100 μl of 0.5 N H_2_SO_4_. The readout was measured at a wavelength of 450 nm, and OD_450_ values were normalized and analyzed. All samples were performed in duplicate.

### Expression and Purification of HA

H1 CA09, H3 HK68, and H3 VIC11 HA constructs C-terminally fused to a thrombin cleavage sequence, T4 fibritin trimerization motif followed by hexahistidine affinity tag were synthesized (Genscript) and subsequently cloned into a pVRC8400 expression plasmid, as previously described (12). HA proteins were expressed by transfection in 293F cells (Thermo Fisher) using Turbo293 transfection reagent (SPEED BioSystem) according to the manufacturer’s protocol. Transfected cells were incubated in shaker incubators at 120 rpm, 37°C, 9% CO_2_ overnight. On the second day, one tenth culture volume of CellBooster medium (ABI scientific) was added to each flask of transfected cells. Cell cultures were then incubated at 120 rpm, 37°C, 9% CO_2_ for an additional 5 days. 6 days post-transfection, cell culture supernatants were harvested, clarified by centrifugation at 2,000 × g and filtered. The supernatants were loaded on Complete His-Tag Resin (Roche) by gravity flow. The resin was washed with three column volumes of PBS with 50 mM imidazole (Roche) and the target protein was subsequently eluted in three column volumes of PBS with 300 mM imidazole. The eluted protein was concentrated and further purified on a Superdex 200 16/60 size exclusion column (GE Healthcare) in PBS.

### Production of Influenza Antibodies

Immunoglobulin heavy chain or light chain sequences were constructed by gene synthesis and then cloned into human IgG1, lambda, or kappa expression plasmids as previously described (12, 59). Heavy and light chain expression plasmid DNA was transfected into Expi293F cells (Thermo Fisher) in 1:1 (v/v) ratio using Turbo293 transfection reagent (60). Monoclonal antibodies from the culture supernatants were purified using recombinant Protein-A Sepharose (GE Healthcare) as per the manufacturer’s instructions.

### Antibody Fab Preparation

The purified human IgG proteins were cleaved by LysC enzyme (1:4000 w/w) (Roche) at 37°C overnight to yield Fabs. On the next day, the enzymatic digestion reaction was terminated by addition of protease inhibitor (Roche). The cleavage mixture was then passed through a protein A column to separate the Fc fragments from the Fab. The Fab collected in the flow-through was loaded onto a Superdex 200 16/60 column for further purification to be used for structure determination.

### Cryo-EM Structure Determination

The H3N2 hemagglutinin (A/Victoria/361/2011) HA trimer was incubated with a molar excess of Fab SRR2899884.46167H+MEDI8852L and 2.3 μl of the complex at 1 mg/ml concentration was deposited on a C-flat 1.2/1.3 carbon grid (protochip.com). The grid was then vitrified using an FEI Vitrobot Mark IV with a wait time of 30 seconds, blot time of 3 seconds, blot force of 1 and humidity of 100%. Data collection on a Titan Krios was performed through Leginon (61) equipped with a Gatan K2 Summit direct detection device. Exposures were collected in movie mode for a 10 s with the total dose of 71.06 e–/Å^2^ fractionated over 50 raw frames. Pre-processing was performed through Appion (62, 63); frames were aligned and dose-weighted with MotionCor2 (64). CTFFind4 (65, 66) was used to estimate the CTF and DoG Picker (62, 63) was used for particle picking, RELION (67) was then used for particle extraction. CryoSPARC 2.15 (68) was subsequently used for the remaining processing of 2D classifications, ab initio 3D reconstruction, homogeneous refinement, and nonuniform 3D refinement. Initial 3D reconstruction was performed using C1 symmetry, confirming 3 Fab molecules per trimer, whereupon C3 symmetry was applied for the final reconstruction and refinement. Model building through coot was followed by simulated annealing and real space refinement in Phenix (69) and then iteratively improved with manual fitting of the coordinates in Coot (70). Geometry and map fitting were evaluated using Molprobity (71) and EMRinger (72). PyMOL (www.pymol.org) and chimera (73) were used to generate figures.

### Pseudotyped Neutralization Assay

Influenza HA-NA pseudotyped lentiviruses that harbor a luciferase reporter gene were produced as described previously (74, 75). Pseudovirus was produced by transfection of 293T cells of HA and corresponding NA along with the lentiviral packaging and reporter plasmids. For all pseudoviruses except H5N1, a human type II transmembrane serine protease TMPRSS2 gene was also contransfected for proteolytic activation of HA to HA1/HA2. Cells were transfected overnight by use of Fugene6 (Promega, Madison, WI) and then replaced with fresh medium. Forty-eight hours after transfection, supernatants were harvested, filtered through a 0.45- μm syringe filter and frozen at -80°C before use.

Neutralization assays were carried out as follows: pseudovirus was mixed with serial dilutions of monoclonal antibodies for 45 minutes followed by addition to 293A cells (Thermo Fisher Scientific) in 96-well plate white/black isoplates (PerkinElmer, Waltham, MA) in triplicate. Three days after infection, cells were lysed in 20μl of cell culture lysis buffer (Promega, Madison, WI) and 50 μl of luciferase assay reagent (Promega) was added. Luciferase activity was measured according to relative light unit (RLU) by MicroBeta luminescence counter (PerkinElmer). IC50 were generated using Prism 8 (GraphPad, San Diego, CA).

### Neutralization Assay Using Engineered Reporter Viruses

Influenza neutralization assay using replication-restricted reporter(R^3^) influenza viruses was described previously (76). Briefly, R^3^ ΔPB1 influenza viruses, which have PB1 coding sequence replaced with TdKatushka2S fluorescent reporter, were rescued by reverse genetics and propagated in PB1-expressing MDCK-SIAT1 cells. For neutralization assay, 4-fold serial antibody dilutions with a starting concentration of 25 ug/ml were made in OptiMEM (Thermo Fisher) supplemented with TPCK-trypsin and incubated with pre-titrated viruses for 1h at 37°C. After incubation, antibody-virus mixtures were transferred in quadruplicates to 384-well plates (Greiner) and 3 ×10^3^ MDCK-SIAT1-PB1 cells were added to each well. Plates were incubated for 18-20h at 37°C in a humidified 5% CO_2_ atmosphere. The number of fluorescent cells in each well was obtained using a Celigo image cytometer (Nexcelom Biosciences) with customized red channel to enhance detection of mKate2/TdKatushka2 reporter (EX 540/80 nm, DIC 593 nm and EM 593/LP nm). The percent neutralization was calculated by constraining the VC control as 0% and the CC control as 100% and plotted against antibody concentration. A curve fit was generated by a four-parameter nonlinear fit model in Prism (GraphPad). The 50% (IC_50_) inhibitory concentrations were obtained from the curve fit for each antibody.

### Homology Modeling and Comparison of Paired Heavy : Light Antibody

Publicly available paired heavy- and light- chain datasets were downloaded using accession numbers from references (77–79). Deep sequencing reads were filtered and sieved by the HV6-1 class signatures. The CDR-H3 sequences were clustered as described in section 2.1, and the centroid antibody of each cluster was selected for modeling of HA/antibody complex structures. Germline amino acids were used to repair the missing residues at N-termini of heavy and light chains. Repaired antibody sequences were threaded on 56.a.09/HA complex structure (PDB:5K9K) (9) and MEDI8852/HA complex structure (PDB:5JW4) (56) structures respectively using software NEST (80). Binding energy between antibody and influenza A virus HA was predicated by Rosetta package, Interface Analyzer, using default parameter (81). The average of the binding energies calculated from the two homology models were used to compare antibodies that satisfied the signatures versus antibodies that satisfied the version 1 signature but not the version 2 signature; two-tailed Mann-Whitney test were used to compare the statistical significance.

### Binding affinity Measurement by Bio-Layer Interferometry

The hexahistidine affinity tagged CA09 or PR34 HA proteins (30 μg/ml) diluted in PBS was captured on Ni-NTA sensor tips to a level of approximately 1.0 – 1.2nm. The duration of protein capture was 300 s. The sensor tips were then washed with PBS for 60 s to allow baseline adjustment. After that, the sensor tips were dipped into the Fabs with eight different concentrations (400 nM, 200 nM, 100 nM, 50 nM, 25 nM, 12.5 nM, 6.25 nM, 0 nM) for an association time of 300 s. Subsequent to Fab binding, the sensors were placed back into PBS for dissociation for 300 s. Antibody binding experiment was performed on a ForteBio OcteRed 96 machine. Subsequent data analysis was performed using the ForteBio Data Analysis 12.0 software.

## Results

### Workflow to Delineate the Sequence Signature of HV6-1 Class Influenza Antibodies

**Figure 1:**
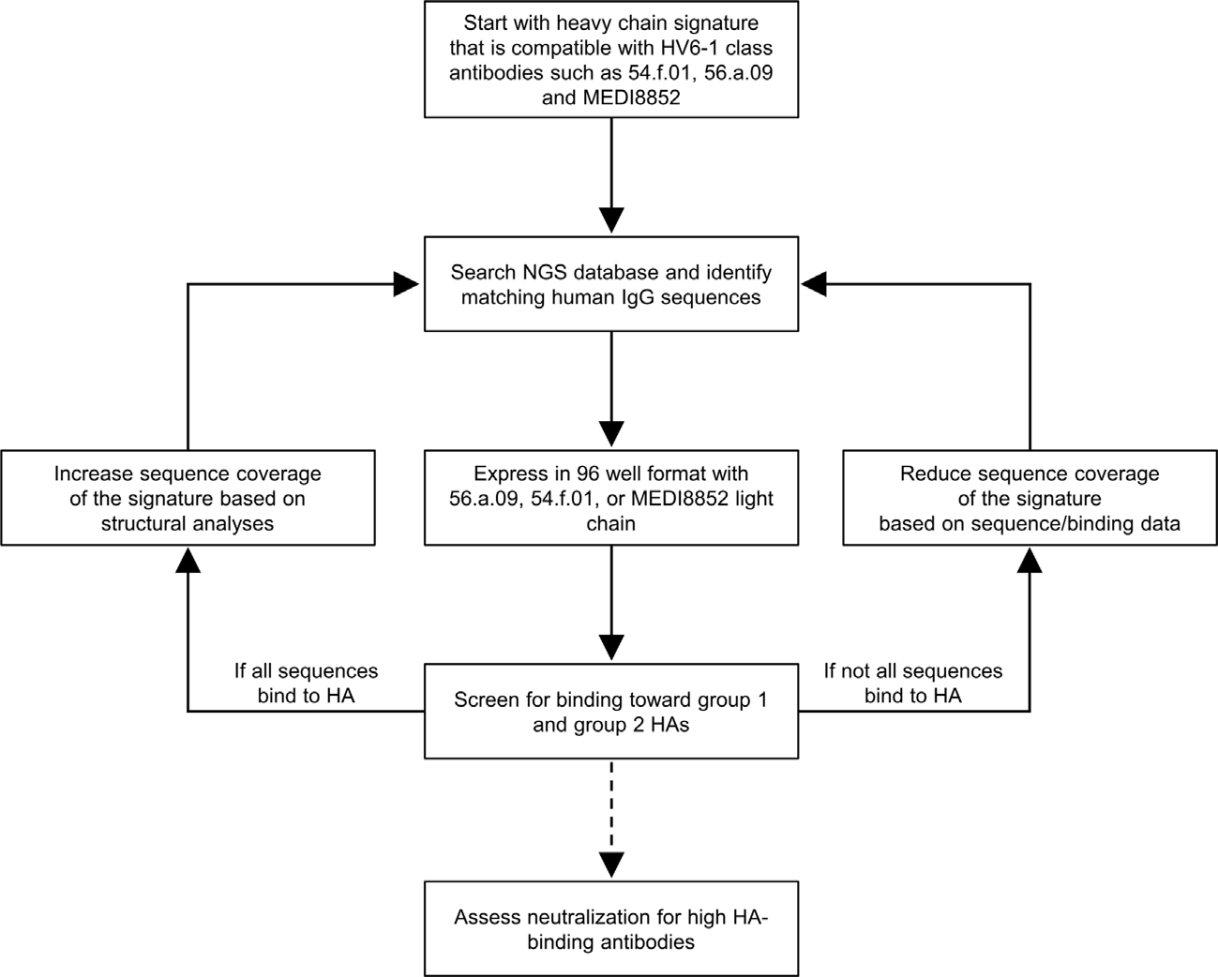
Iterative workflow to improve the sequence signature-based identification of human HV6-1 class influenza antibodies. In this protocol, a starting sequence signature that is compatible to all known HV6-1 class influenza antibodies was used to screen against published NGS databases of human IgG sequences, comprise mostly unpaired sequences of IgG-heavy chains or IgG-light chains. The heavy chain sequences that satisfied the sequence signature were paired with light chains from known HV6-1 class influenza antibodies respectively and assessed for their binding toward group 1 and group 2 hemagglutinins (HAs). The sequence signature can be further optimized based on the proportion of the signature-identified antibodies that are functional. Select antibodies with high HA-binding were assessed for their neutralization capacities.

We developed a workflow to delineate antibody sequence signature in general and applied it to delineate the sequence signature of HV6-1 class influenza antibodies (**Figure 1**). Briefly, we start with a broader signature that are able to accommodate all known HV6-1 class influenza antibodies, use this sequence signature to identify antibody sequences from sequence databases, and then characterize their binding to hemagglutinin. As we only defined the signature for heavy chain, we pair the identified heavy chain sequences with three known HV6-1 antibodies: MEDI8852, 56.a.09, and 54.f.01. If all or majority of the sequences are functional, the sequence signature would be made broader and the sequence search and binding analyses would be repeated again. If substantial number of the sequences are not functional, this would suggest the sequence signature is too broad, and the sequence signature would be made more restrictive by adding amino acid requirements based on the informatics analyses from the binding data. Select number of antibodies would also be selected to assess for their neutralization against group 1 and group 2 viruses.

### HV6-1 Class Signature Version 1 Identified 22 Functional and 40 Non-Functional Heavy Chain Sequences From NGS Dataset

**Figure 2:**
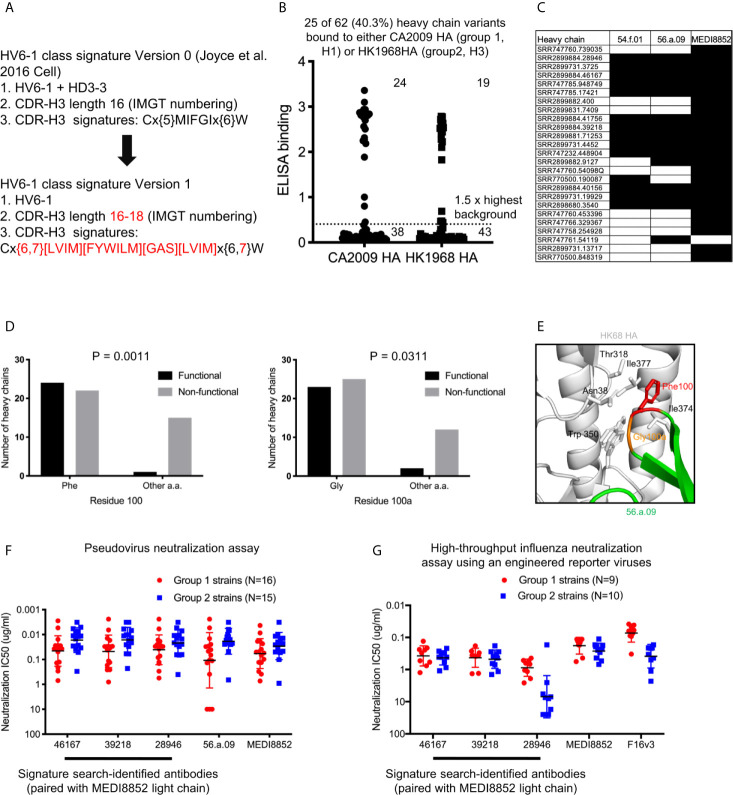
Sequence signature optimization and antibody identification for HV6-1 class antibody based on sequence search of NGS database of human IgG-heavy chain sequences and antigenic screening. **(A)** HV6-1 class signature version 1, with the modification from the original sequence signature published in Joyce et al. Cell 2016 highlighted in red. **(B)** ELISA binding of 62 HIV6-1 class signature version 1-identified heavy chain variants to hemagglutinin of CA2009 or HK1968, when paired with light chain of 56.a.09, 54.f.01, or MEDI8852 (highest value of the three were displayed). **(C)** Light chains that resulted in functional antibodies when paired with the signature-identified heavy chains. Heavy chain entries that were functional when paired at least of the three light chains are shown. Heavy/light chain pairing that resulted in functional antibodies (e.g. with ELISA signal greater than 1.5 fold of the maximum background for either CA2009 or HK1968 HA) are shown in black fill. **(D)** 2x2 contingency analysis of the functionality of signature-identified heavy chain sequence versus the presence of phenylalanine at residue 100 or glycine at residue 100a, respectively. P-values were calculated using two-tailed Fisher’s Exact test. **(E)** Close up view of residues F100 and G100a in a hemagglutinin structure in complex with antibody 56.a.09 (PDB:5K9K). **(F)** Neutralization IC80 of three HV6-1 class signature version 1-identified heavy chains paired with MEDI8852 light chain when assessed with a pseudovirus assay. 46167 is short for SRR2899884.46167. 39218 is short for SRR2899884.39218. 28946 is short for SRR2899884.28946. **(G)** Neutralization IC50 of three HV6-1 class signature version 1-identified heavy chains paired with MEDI8852 light chain when assessed with a high-throughput influenza neutralization assay using engineered reporter viruses.

As opposed to the HV6-1 class influenza antibody signature initially described in Joyce et al., 2016 (termed HV6-1 class signature version 0 in this paper), which is not compatible with other known HV6-1 antibodies such as MEDI8852, we started with a broader sequence signature in this paper (HV6-1 class signature version 1, **Figure 2A**). Specifically, we specified the CDR-H3 length to be 16-18 amino acids long as opposed to limiting the CDR-H3 length to only 16 amino acids long as in the version 0 signature, as 56.a.09 had a CDR-H3 length of 16 amino acids while MEDI8852 had a CDR-H3 length of 18 amino acids. In addition, version 1 signature allows full amino acid flexibility at residue 98, and allowing amino acid flexibility limited to similar residue types for the ^99^IFGI motif in version 0 signature, as residue 98 did not contact hemagglutinin for both 56.a.09 and MEDI8852 (**Figure S1**). In addition, we removed the requirement of HD3-3 germline gene as D gene determination can be sometimes ambiguous. We used the version 1 signature to search for heavy chain sequences from three NGS datasets from three influenza vaccination three studies (**Table S1A**), and identified a total of 62 cluster representatives (**Figure S2A**, **Table S1**, and **Dataset S1**). We co-expressed the heavy chains with three different light chains from known HV6-1 class antibodies respectively, including MEDI8852, 56.a.09, and 54.f.01, and assessed the binding to hemagglutinin of A/California/04/2009 (CA2009, H1 subtype) and H3 A/Hong Kong/1/1968 (HK1968, H3 subtype) using a 96-well transient expression ELISA assay (see Methods). We observed that 24 of the 62 heavy chain sequences, when paired with one of the three HV6-1 class antibody light chains, have high binding towards CA2009 HA, and 19 of the 62 heavy chains, when paired with one of the three HV6-1 class antibody light chains, have high binding toward HK1968 HA (see Methods) (**Figure 2B**, **Dataset S2**). Overall, 25 of the 62 heavy chains (40.3%), when paired with one of the three HV6-1 class antibody light chains, have high binding toward CA2009 HA or HK1968 HA. Specifically, 24 of these 25 heavy chains are functional when paired with MEDI8852 light chain (KV1-39 derived), while 16 of these 25 heavy chains are functional when paired with 56.a.09 or 54.f.01 light chain (KV3-20 derived) (**Figure 2C**).

As majority of the version 1 sequence signature-identified heavy chains were not functional, we examined the association between amino acid types at each residue position and functional outcome. We observed that 23 of the 25 functional heavy chains had a phenylalanine at residue 100 (P=0.0011) and 22 of the 25 functional heavy chains had a glycine at residue 100a (P=0.0311) (**Figure 2D**). Examination the structure of antibody 56.a.09 in complex with hemagglutinin revealed that Phe100 binds to a cavity lined by hydrophobic residues such as Trp350, Ile372, and Il377 (PDB:5K9K) (**Figure 2E**). We also noticed that among the 62 antibodies, larger fraction of antibodies with CDR-H3 length of 17 were functional compared to antibodies with CDR-H3 length of 16 or 18 **(** **Figure S3**), suggesting that CDR-H3 length of 17 is compatible with HV6-1 class antibody signature.

### HV6-1 Class Signature Version 1 Identified Antibodies With Comparable Neutralization Potency and Breadth to MEDI8852

To evaluate if the signature-identified HV6-1 class antibodies can neutralize influenza viruses in addition to binding to hemagglutinin, we evaluated entry-inhibition for 10 of the 22 heavy chains that, when paired with one of the three known HV6-1 class antibody light chains, showed the highest binding to hemagglutinin, using a pseudovirus assay with a panel of six influenza strain (**Dataset S3**). All ten tested antibodies inhibited the entry of all six viruses. Next, we selected the top three antibodies based on the results of the six strain panel, and evaluated their entry inhibition against a panel of 31 influenza strains, including 16 group 1 strains and 15 group 2 strains (**Figure 2F**, **Dataset S4**), and observed that all three signature-identified heavy chains, when paired with the light chain from MEDI8852, showed comparable neutralization potency and breadth to MED8852. We also assessed the neutralization of these three signature-identified antibodies with a high-throughput influenza neutralization assay using engineered reporter viruses (see methods), and showed that two of these three antibodies (SRR2899884.46167 and 39218, paired with MEDI8852 light chain respectively) showed comparable neutralization potency and breadth to MEDI8852 (**Figure 2G** and **Dataset S5**).

### SRR2899884.46167H+MEDI8852L Showed Similar CDR-H3 Recognition of Hemagglutinin as MEDI8852 and 56.a.09

**Figure 3:**
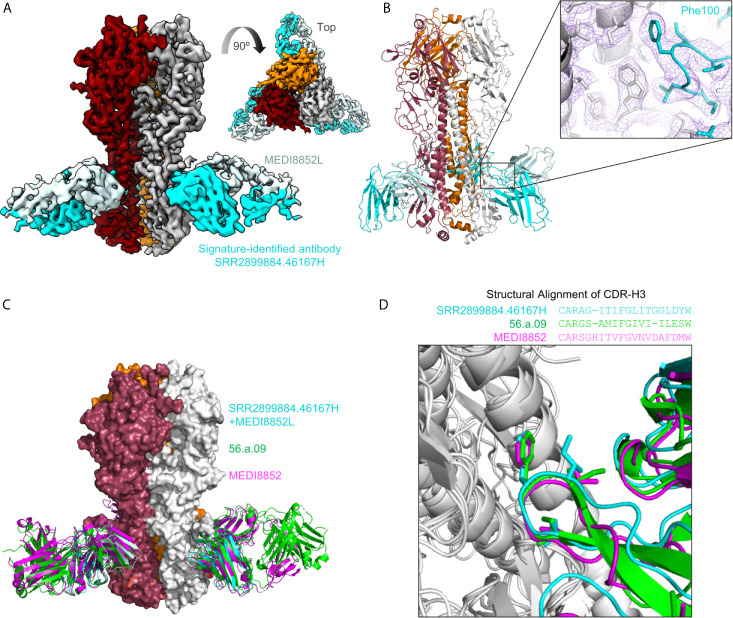
Cryo-EM structure of signature-identified antibody SRR2899884.46167H+MEDI8852L in complex with hemagglutinin, confirms similar modes of recognition. **(A)** 3D reconstruction of SRR2899884.46167H+MEDI8852L in complex with HA trimer at 3.4 Å. **(B)** Cartoon representation of the complex. Inset shows quality of density surrounding the Phe100 interaction. **(C)** Overlay of 56.a.09 (PDB:5K9K, green), MEDI8852 (PDB:5JW4, magenta), and signature identified antibody SRR2899884.46167H+MEDI8852L in complex with HA. **(D)** Sequence alignment of CDR-H3 based on structure is shown above an overlay of CDR-H3 from the three antibodies that highlights similar CDR-H3 recognition.

To examine if the sequence-signature-identified HV6-1 sequences have similar recognition mode as known HV6-1 antibodies, we solved the structure of SRR2899884.46167H+MEDI8852L in complex with an H3N2 hemagglutinin (A/Victoria/361/2011) using cryo-EM at 3.41 angstroms (**Figures 3A, B** and **S4**). When the structures of hemagglutinin in complex with MEDI8852 (PDB:5JW4) and 56.a.09 (PDB:5K9K) were aligned on top of this structure based on hemagglutinin (**Figure 3C**), the ^99^VFGV^100b^ motif of MEDI8852 CDR-H3, the ^99^IFGI^100b^ motif of 56.a.09 CDR-H3, and ^99^IFGL^100b^ motif of SRR2899884.46167 CDR-H3 align almost perfectly on top of each other (RMSD<1 Å, **Figure 3D**), demonstrating that the signature-search-identified HV6-1 heavy chain SRR2899884.46167 can recognize hemagglutinin in the similar manner as previously identified HV6-1 class antibodies.

### HV6-1 Class Signature Version 2 Identified Functional HV6-1 Class Antibodies With Improved Accuracy

**Figure 4:**
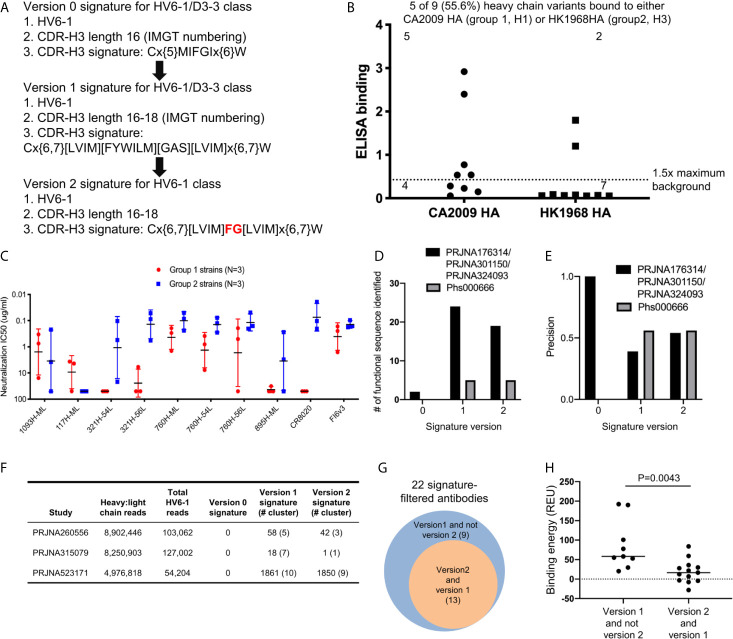
Next generation HV6-1 antibody class signature (version 2) can identify functional HV6-1 class antibodies with improved accuracy. **(A)** HV6-1 class signature version 2, with the modification from HV6-1 class signature version 1 highlighted in red. **(B)** ELISA binding of 9 HV6-1 class signature version 2-identified heavy chain variants to hemagglutinin of CA2009 or HK1968, when paired with light chain of 56.a.09, 54.f.01, or MEDI8852. **(C)** Neutralization IC_50_ of five HV6-1 class signature version 2-identified heavy chains paired with select light chains when assessed with a pseudovirus assay. **(D)** Number of functional sequences identified by each of the three signatures from the first (PRJNA176314/PRJNA301150/PRJNA324093) and second (Phs000666) sequence database search. **(E)** Precision (defined as TP/(TP+FP)) for identification of functional sequences for the three signatures from the first and second sequence database search. TP, true positive; FP, false positive. **(F)** Number of reads, and number of HV6-1 class influenza antibodies in paired heavy:light chain BCR sequencing. **(G)** Venn diagrams showing overlaps of antibodies selected by signature version 1 and version 2. The number inside parentheses indicates the number of antibody clusters. **(H)** Comparison of binding energy (*in silico* calculation) to hemagglutinin between antibodies selected by version 1 but not version 2 signature (left) and antibodies compatible with both version 1 and version 2 signatures (right). Two-tailed Mann-Whitney test was used for statistical comparison.

Based on the antigenic and bioinformatics analyses of the sequences identified from HV6-1 class signature version 1 (**Figure 2**), we came up with the version 2 of HV6-1 class signature by allowing only phenylalanine at residue 100 and glycine at residue 100a (**Figure 4A**). We used the HV6-1 class signature version 2 to search for HV6-1 class sequences from another NGS dataset (dbGaP Study Accession: phs000666.v1.p1), which had no sequence overlap to the NGS datasets used in the prior search. Sixteen out of 1,611,992 productive heavy sequences were compatible with the signature version 2. After clustering, we co-expressed nine heavy chains (**Figure S2B**, **Dataset S6A**) with light chains from MEDI8852, 56.a.09, and 54.f.01, respectively, and assessed the binding to hemagglutinin of A/California/04/2009 (CA2009, H1 subtype) and H3 A/Hong Kong/1/1968 (HK1968, H3 subtype) using a 96-well transient expression ELISA assay (see Methods). We observed that five of the nine heavy chain sequences (56%), when paired with one of the three HV6-1 class antibody light chains, have high binding towards CA2009 HA, and two of these nine heavy chains, when paired with one of the three HV6-1 class antibody light chains, have high binding toward HK1968 HA (see Methods) (**Figure 4B**, **Dataset S7**). We assessed entry-inhibition of eight antibodies, including five unique heavy chains, with the highest ELISA signal using three group 1 and three group 2 viral strains. All eight antigenic-positive antibodies showed activity towards at least two different strains (**Figure 4C**, **Dataset S8**), confirming the signature version 2-identified antibody sequences to be functional in terms of neutralization. Notably, version 0 signature did not identify any reads, and version 1 signature identified the same set of sequences as identified by version 2 signature (**Table S1A**).

In addition, we used versions 0, 1, and 2 HV6-1 class antibody signatures to search for paired heavy and light chain sequences from three different deep sequencing samples (**Figure 4F**). We found no sequences satisfying version 0 signature and 22 sequence cluster representatives satisfying version 1 signature, among which 13 also satisfying version 2 signature (**Figure 4F**). Nine different light chain germline genes were observed among the 22 sequence cluster representatives from version 1 signature search, including KV1-39 and KV3-20 used by MEDI8852 and 56.a.09, respectively (**Dataset S6B**); version 2-selected antibodies derived from 8 different light chain germline genes and showed an increase in the fraction from KV-gene origin (**Dataset S6B**). We constructed homology model for the 22 antibodies, analyzed the predicted binding energy between Flu HA and antibody, and observed the 13 antibodies satisfying the version 2 signature to have lower binding energy than the 9 antibodies satisfying version 1 but not the version 2 signature (**Figures 4G, H**).

Finally, we used versions 0, 1, and 2 HV6-1 class antibody signatures to search NGS samples of Naïve B cells and cord blood (78, 82), and found that while there are no sequences that satisfied version 0 signature, sequences that satisfied versions 1 and 2 signature were present in these NGS reads (**Table S1B**), suggesting that antibodies with HV6-1 class antibodies characteristics are present before immunization.

## Discussion

In this study we developed a pipeline to optimize antibody sequence signatures based on iterative sequence search and antigenic assessment. Specifically, we optimized the sequence signature of HV6-1-class influenza antibodies and identified new antibody members of the class by searching NGS dataset with sequence signatures. We also showed the 46167H/MEDI8852L antibody, of which the heavy chain was identified from the sequence signature search, to neutralize diverse strains of influenza A and to recognize hemagglutinin in a manner similar to that of MEDI8852 and 56.a.09, despite having differences in CDR-H3 length.

Comparing the prediction performances of different HV6-1 signature versions, we noticed that the version 1 and 2 signatures had higher sensitivity in identifying functional HV6-1 class antibodies as compared to the version 0 signature (**Figure 4D**, **Table S1A**), and the version 2 signature had higher precision than version 1 (**Figures 4E–G**, **Table S1A**). We were, however, unable to assess the false negative rate of our search, as we could not assess the function of all antibodies derived from HV6-1 germline gene, which are in the order of hundreds of thousands per donor (**Table S1C**).

Another caveat of the study was that most of the sequencing datasets we examined only had heavy chain sequences, not paired heavy-light chain sequences. but in general either did not find heavy chain sequences that matched the HV6-1-sequence signature, or the light chain sequences were incomplete. We did search on single cell sequencing datasets of influenza vaccination, healthy and treatment of disease for HV6-1 signature matched sequences (PMID:33287869, 32573488, 32866963); however, zero reads matched HV6-1-sequence signature because of low number of IGHV6-1 germline sequences in recovered cells. We paired heavy chains we identified from the sequence signature search with light chains from three of the known HV6-1 class antibodies, which could result in increased false negative rate as the heavy chains were not paired with their native light chain.

Notably, we observed that reverting the light chain residues to germline version generally did not reduce binding affinity by more than three-fold, while reverting the heavy chain residues, or residues from both chains, to germline had a much greater impact (**Figure S5**). In addition, while these antibodies employed light chains in binding to hemagglutinin, their contribution to binding are expected to be lower than that of the heavy chain, based on binding interface area calculation (the light chains contributed 40% and 27% to the total protein buried surface area for 56.a.09 and MEDI8852, respectively). Therefore, in the absence of pairing with functional HV6-1 heavy chains, the antibody is unlikely to be active and its binding affinity to hemagglutinin is likely to be too low to be detected by ELISA.

We also note that in a recently published study two clonal types of HV6-1 antibodies, 54-1G05 and 54-4H03, were isolated in a single donor (31), but only the 54-1G05 clonal type is compatible with the sequence signatures developed here. Further investigation is needed to develop a sequence signature that would encompass the other distinct clonal type from that study, 54-4H03, which utilizes a divergent CDR-H3 mode of recognition, which can nonetheless evolve to neutralize both group 1 and group 2 strains of influenza A virus. In general, antibodies evolve diverse ways to recognize similar epitopes, with this diversity contending with multidonor class reproducibility. This contention, between diversity and reproducibility is mirrored by the completeness and accuracy of class-base sequence signatures.

Overall, the results suggest that the workflow developed in this study can nonetheless be useful in improving the sequence signature for multidonor antibody classes – both to revise the signature to encompass more divergent class members, such as was carried out here to include MEDI8552 in this study, or to include 54-4H03, in a future study. It will be interesting to see how improved HV6-1 class signatures can be integrated into the development of class-based immunogens, which seek to elicit broad humoral immunity against diverse influenza A viral strains.

## Data Availability Statement

NGS datasets analyzed in this study (accession numbers listed in **Figure 4F**, **Tables S1, S2**) can be downloaded from the Short Read Archive server (https://www.ncbi.nlm.nih.gov/sra). EM map and coordinates for the structure of SRR2899884.46167H+MEDI8852L in complex with A/Victoria/361/2011 hemagglutinin have been deposited to Electron Microscopy Data Bank under accession code EMD-22804, and to Protein Data Bank under accession code 7KC1, respectively.

## Author Contributions

G-YC and PK designed the experiments. G-YC, C-HS, and RR performed the informatics analyses. CC, MJ, BZ, and TZ expressed and produced the antibodies. JG determined the cryo-EM structure. AC, MK, and BG performed influenza neutralization assay using engineered reporter viruses. KL, LW, EY, YZ, and JM performed pseudo-typed neutralization assay. YY performed 96-well ELISA screening. BD identified NGS datasets for analyses. G-YC, C-HS, and JG generated the figures and tables. G-YC and PK wrote the manuscript. All authors contributed to the article and approved the submitted version.

## Funding

Support for this work was provided by the Intramural Research Program of the Vaccine Research Center, National Institute of Allergy and Infectious Diseases, National Institutes of Health. Some of this work was performed at the Simons Electron Microscopy Center and National Resource for Automated Molecular Microscopy located at the New York Structural Biology Center, supported by grants from the Simons Foundation (SF349247), NYSTAR, and the NIH National Institute of General Medical Sciences (GM103310).

## Conflict of Interest

The authors declare that the research was conducted in the absence of any commercial or financial relationships that could be construed as a potential conflict of interest.
