# Supplementary material for: Sequence-Signature Optimization Enables Improved Identification of Human HV6-1-Derived Class Antibodies That Neutralize Diverse Influenza A Viruses
Source: Front Immunol. 2021 May 31;12:662909. doi: 10.3389/fimmu.2021.662909 (PMC8201785; doi:10.3389/fimmu.2021.662909)
Supplement: Supplementary file 1 [file DataSheet_1.pdf]

Dataset S1. Sixty-two heavy chain sequences identified from sequence signature search using HV6-1 class signature version 1. Missing residues were filled in with germline amino acids.

SRR3620045.166398	QVQLQQSGPGLVKPSQTLSLTCAISGDSVSSNSAAWNWIRQSPSRGLEWLGRTYYRSKWYNDYAVSVKSRITINPDTSKNQFSLQLNSVTPEDTAVYYCARGRRVTIFGVVTHWFDPWGQGTLVTVSS
SRR3620045.141882	QVQLQQSGPGLVKPSQTLSLTCAISGDSVSSNSAAWNWIRQSPSRGLEWLGRTYYRSRWFNDYAASMRSRIIIDSDTSTNRLSLQLNSVTPEDTAIYYCASS-NIPLFGVDSNWFDAWGQGTLVTVSS
SRR3620050.863624	QVQLQQSGPGLVMPSETLSLTCAISGDSVSSNSAAWNWIRQSPSRGLEWLGRTYYRSKWYNDYAVSVKSRITINPDTSKNQFSLQLNSVTPEDTAVYYCARD-RFYVWGID-PYFDYWGQGTLVTVSS
SRR770500.848319	QVQLQQSGPGLVKPSQTLSLTCAISGDSVSSNSAAWNWIRQSPSRGLEWLGRTYYRSKWYNDYAVSVKSRITINPDTSKNQFSLQLNSVTPEDTAVYYCARAYIVVVIAIGWDAFDIWGQGTMVTVSS
SRR747232.465310	QVQLQQSGPGLVKPSQTLSLTCAISGDSVSSNSAAWNWIRQSPSRGLEWLGRTYYRSKWYNDYAVSVKSRITINPDTSKNQFSLQLNSVTPEDTAVYYCARD-QWFLWAVAERGFDYWGQGTLVTVSS
SRR747758.254928	QVQLQQSGPGLVKTSQTLSLTCAISGDSVSSNSAAWNWIRQSPSRGLEWLGRTYYRSKWYNDYAVSVKSRITINPDTSKNQFSLQLNSVTPEDAAVYYCARA-TITIFGVVTYWFDPWGQGTLVTVSS
SRR747760.344508	QVQLQQSGPGLVKPSQTLSLTCAISGDSVSSNSAAWNWIRQSPSRGLEWLGRTYYRSKWYNDYAVSVKSRITINPDTSKNQFSLQLNSVTPEDTAVYYCARVPTVIIFGVV-NFFDYWGQGTLVTVSS
SRR747760.959110	QVQLQQSGPGLVKPSQTLSLTCAISGDSVSSNSAAWNWIRQSPSRGLEWLGRTYYRSKWYNDYAVSVKSRITINPDTSKNQFSLQLNSVTPEDTAVYYCARTDRNTIFGVE-TEFDYWGQGTLVTVSS
SRR747760.449420	QVQLQQSGPGLVKPSQTLSLTCAISGDSVSSNSAAWNWIRQSPSRGLEWLGRTYYRSKWYNDYAVSVKSRITINPDTSKNQFSLQLNSVTPEDTAVYYCAREAKSLVLGLG-WYFDLWGRGTLVTVSS
SRR747760.949949	QVQLQQSGPGLVKPSQTLSLTCAISGDSVSNNRAAWSWIRQSPLRGLEWLGRTYYMSKWYHEYGVSVKGRITINPDTSKNQFSLQLNSVTPEDTAVYYCARD-QPLILSVLEYYFDYWGQGTLVTVSS
SRR747760.54098	QVQLQQSGPGLVKPSQTLSLTCAISGDSVSSNSAAWNWIRQSPSRGLEWLGRTYYRSKWYNDYAVSVKSRITINPDTSKNQFSLQLNSVTPEDTAVYYCAREY-STIFGVVIRMFDYWGQGTLVTVSS
SRR747760.676398	QVQLQQSGPGLVKPSQTLSLTCAISGDSVSSNSAAWNWIRQSPSRGLEWLGRTYYRSKWYNDYAVSVKSRITINPDTSKNQFSLQLNSVTPEDTAVYYCARG-GITIFGVANNWFDPWGQGTLVTVSS
SRR747760.453396	QVQLQQSGPGLVKPSQTLSLTCAISGDSVSSNSAAWNWIRQSPSRGLEWLGRTYYRSKWYNDYAVSVKSRITINPDTSKNQFSLQLNSVTPEDTAVYYCARDQSITIFGVA-RSFDYWGQGTLVTVSS
SRR747760.571908	QVQLQQSGPGLVKPSQTLSLTCAISGDSVSSNSAAWNWIRQSPSRGLEWLGRTYYRSKWYNDYAVSVKSRITINPDTSKNQFSLQLNSVTPEDTAVYYCARVHVSSVIAVAGTGFDPWGQGTLVTVSS
SRR747760.757256	QVQLQQSGPGLVKPSQTLSLTCAISGDSVSSNSAAWNWIRQSPSRGLEWLGRTYYRSKWYNDYAVSVKSRITTNPDTSKNQFSLQLNSVTPEDTAVYYCAGE-RAGIFGVVNGPIDYWGQGTLVTVSS
SRR747760.1092354	QVQLQQSGPGLVKPSQTLSLTCAISGDSVSSNSAAWNWIRQSPSRGLEWLGRTYYRSKWYNDYAVSVKSRITINPDTSKNQFSLQLNSVTPEDTAVYYCAREAQITIFGVV-TYFDYWGQGTLVTVSS
SRR747766.762496	QVQLQQSGPGLVKPSQTLSLTCAISGDSVSSNSAAWNWIRQSPSRGLEWLGRTYYRSKWYNDYAVSVKSRITINPDTSKNQFSLQLNSVTPEDTAVYYCARSPPEDLIAVA-GYFDYWGQGTLVTVSS
SRR747766.329367	QVQLQQSGPGLVKPSQTLSLTCAISGDSVSSNSAAWNWIRQSPSRGLEWLGRTYYRSKWYNDYAVSVKSRITINPDTSKNQFSLQLNSVTPEDTAVYYCAKN-SLTIFGVVSSGMDVWGQGTTVTVSS
SRR747768.239777	QVQLQQSGPGLVKPSQTLSLTCAISGDSVSSNSAAWNWIRQSPSRGLEWLGRTYYRSKWYNDYAVSVKSRITINPDTSKNQFSLQLNSVTPEDTAVYYCAPRKYDYVWGVK-VSCDYWGQGTLVTVSS
SRR747785.948749	QVQLQQSGPGLVKPSQTLSLTCAISGDSVSSDSGAWNWIRQSPSRGLEWLGRTYYRSRWYNDYAVSVKSRIVINSDTSKNQLSLQLKSVTPEDTAVYYCARS-GVKIFGIVVMALDYWGQGSPVTVSS
SRR747785.17421	QVQLQQSGPGLVKPSQTLSVTCAISGDSVSSNSAAWNWVRQSPSRGLEWLGRTYYRSRWYNDYAASVKSRIIINQDTSKNQFSLQLKSVTPEDTAVYYCARA-GVRIFGIIIMALDYWGQGTLITVSS
SRR747785.35227	QVQLQQSGPGLVKPSQTLSLTCAISGDSVSTNSAAWNWIRQSPSRGLEWLGRTYYRSKWYNDYAVSVKSRITINPDTSKNQFSLQLNSVTPEDTAVYYCSRDQLVTIFGVV-IRNDYWGQGTLVTVSS
SRR770500.554839	QVQLQQSGPGLVKPSQTLSLTCAISGDSVSSNSAAWNWIRQSPSRGLEWLGRTYYRSKWYNDYAVSVKSRITINPDTSKNQFSLQLNSVTPEDTAVYYCART-ASPVIAVARYGMDVWGQGTTVTVSS
SRR770500.190087	QVQLQQSGPGLVKPSQTLSLTCAISGDSVSGNSGAWNWIRQSPSRGLEWLGRTYYRSKWYNDYAESVKSRITINADTSKNQFSLQLKSVTPEDTAVYYCARA-GIMIFGVVRNAFDIWGQGTMVTVSS
SRR770500.48601	QVQLQQSGPGLVKPSQTLSLTCAISGDSVSSNSAAWNWIRQSPSRGLEWLGRTYYRSKWYNDYAVSVKSRITINPDTSKNQFSLQLNSVTPEDTAVYYCARDLSPEIIAVA-GGLDYWGQGTLVTVSS
SRR747760.739035	QVQLQQSGPGLVKPSQTLSLTCAISGDSVSSNSAAWNWIRQSPSRGLEWLGRTYYRSKWYNDYAVSVKSRITINPDTSKNQFSLQLNSVTPEDTAVYYCARDGGATIFGVVILAFDIWGQGTMVTVSS
SRR770500.197074	QVQLQQSGPGLVKPSQTLSLTCAISGDSVSSNSAAWNWIRQSPSRGLEWLGRTYYRSKWYNDYAVSVKSRITINPDTSKNQFSLQLNSVTPEDTAVYYCARV-FAVLIAVAGNWFDPWGQGTLVTVSS
SRR747232.448904	QVQLQQSGPGLVKPSQTLSLTCAISGDSVSSNSVAWNWIRQSPSRGLEWLGRTYHRSRWYNDYAVSLKGRISINPDTSKNLFSLQLKSVTPEDTAVYYCARQ-SPMIFGIV-MTVDYWGPGTVVTVSS
SRR747232.167571	QVQLQQSGPGLVKPSQTLSLTCAISGDSVSSNSVTWNWIRQSPSRGLEWLGRTYHRPRWYNDYAVSLKGRISINPDTSKNLFSLQLKSVTPEDTAVYYCARQ-SPMIFGIV-MTVDYWGPGTVVTVSS
SRR747758.172133	QVQLQQSGPGLVKPSQTLSLTCGISGDSVASNSAAWNWIRQSPSRGLEWLGRTFYRSKWYNHYAGSVRSRITISPDTSKNQFSLQLTSVTPEDTAVYYCARS-RFTVIGVV-TLPDYWGQGTLVTVSS
SRR747760.66232	QVQLQQSGPGLVKPSQTLSLTCAISGDSVSSNSAAWNWIRQSPSRGLEWLGRTYYRSKWYNDYAVSVKSRITINPDTSKNQFSLQLNSVTPEDTAVYYCARE-TQLVWALY-YGMDVWGQGTTVTVSS
SRR747767.310560	QVQLQQSGPGLVKPSQTLSLTCAISGDSVSSNSAAWNWIRQSPSRGLEWLGRTYYRSKWYNDYAVSVKSRITINPDTSKNQFSLQLNSVTPEDTAVCYCAR-GVLMVYAIG-WYFDLWGRGTLVTVSS
SRR747785.850579	QVQLQQSGPGLVKPSQTLSLTCAISGDSVSSKSAAWNWIRQSPSRGLEWLGRTYYRSKWYDDYAVSVQSRITINPDTSKNQFSLQLNSVTPEDTAIYYCARA-STVLIAVA-ATFDYWGQGTLVTVSS
SRR747785.133240	QVQLQQSGPGLVKPSQTLSLTCAISGDSVSSNSAAWNWIRQSPSRGLEWLGRTYYRSKWYNDYAVSVKSRITINPDTSKNQFSLQLNSVTPEDTAVYYCARDEGA-IFGVV-IIFDYWGQGTLVTVSS
SRR747785.830930	QVQLQQSGPGLVKPSQTLSLTCAISGDSVSSNSAAWNWIRQSPSRGLEWLGRTYYRSKWYNDYAVSVKSRITINPDTSKNQFSLQLNSVTPEDTAVYYCARG-SVTIFGV-ESWFDPWGQGTLVTVSS
SRR770500.58736	QVQLQQSGPGLVKPSQTLSLTCAISGDSVSSNSAAWNWIRQSPSRGLEWLGRTYYRSKWYNDYAVSVKSRITINPDTSKNQFSLQLNSVTPEDTAVYYCARDY-SGIFGVVTP-FDYWGQGTLVTVSS
SRR747760.1120790	QVQLQQSGPGLVKPSQTLSLTCAISGDSVSSNSAAWNWIRQSPSRGLEWLGRTYYRSKWYNDYAVSVKSRITINPDTSKNQFSLQLNSVTPEDTAVYYCARDRSLTIFGVVITLFDYWGQGTLVTVSS
SRR747763.163801	QVQLQQSGPGLVKPSQTLSLTCAISGDSVSSNSAAWNWIRQSPSRGLEWLGRTYYRSKWYNDYAVSVKSRVTINPDTSKNEFSLQLNSVTPEDTAVYYCARQQRITIFGVVITAFDYWGQGNLVTVSS
SRR747761.54119	QVQLQQSGPGLVKPSQTLLLTCAISGDSVSSNSAAWNWIRQSPSRGLEWLGRTYYRSKWYNDYAVSVKSRVTINPDTSKNEFSLQLNSVTPEDTAVYYCARQQRITIFGVVITAFDYWGQGNLVTVSS
SRR747766.705555	QVQLQQSGPGLVKPSQTLSLTCAISGDSVSSNTAAWNWIRQSPSRGLEWLGRTYYRSKYYFDYAVSVKSRITISPDTSKNQFSLQLTSVTPEDTAVYYCARGRWWLILSLYYYGVDVWGQGTTVTVSS
SRR747766.1196535	QVQLQQSGPGLVKPSQTLSLTCAISGDSVSSNLVTWNWIRQSPSRGLEWLGRTYYRSKWYNDYAVSVKSRITINPDTSKNQFSLQLNSVTPEDTAVYYCARGLEDTIFGVVTPGFDYWGQGTLVTVSS
SRR747766.318267	QVQLQQSGPGLVKPSQTLSLTCAISGDSVSSNSAAWNWIRQSPSRGLEWLGRTYYRSKWYNDYAVSVKSRITINPDTSKNQFSLQLNSVTPEDTAVYYCARGLGDTIFGVVTPGFDYWGQGTLVTVSS
SRR747767.840275	QVQLQQSGPGLVKPSQTLSLTCAISGDSVSSNSAAWNWIRQSPSRGLEWLGRTYYRSKWYNDYAVSVKSRITINPDTSKNQFSLQLNSVTPEDTAVYYCARDPQVTIFGVVIIYFDYWGQGTLVTVSS
SRR770500.180443	QVQLQQSGPGLVKPSQTLSLTCAISGNSVSSNSAGWNWIRQSPSRGLEWLGRTYYRSKWYNNYAVSVKSRITINPDTSKNQFSLQLNSVTPEDTAVYYCARDYRGSIFGLAPGWFDPWGQGTLVTVSS
SRR747785.739684	QVQLQQSGPGLVKPSQTLSLTCAISGDSVSSNSAAWNWIRQSPSRGLEWLGRTYYRSKWYNDYAVSVKSRITINPDTSKNQFSLQLNSVTPEDTAVYYCARGHRITIFGVVEIPLLSWGQGTLVTVSS
SRR2899731.13717	QVQLQQSGPGLVKPSQTLSLTCAISGDSVSSSSAAWNWLRQSPSRGLEWLGRTYYRSKWYNDYAESVKGRITINPDTSKNQFSLQLNSVTPEDSAFYYCARS-SITIFSLA-SGLDVWGQGTTVTVSS
SRR2899731.31645	QVQLQQSGPGLVKPSQTFSLTCAISGDSVSSNSAAWNWLRQSPSRGLEWLGRTYYRSKWYNDYAESAKGRITINADTSKNQLSLQLNSVTPEDSAFYYCARS-SITIFSLA-SGLDVWGQGDHGHRLL
SRR2898680.3540	QVQLQQSGPGLVKPSQTLSVTCAISGDTVSSNSAAWNWIRQSPSRGLEWLGRTYYRSKWYNDYAVSVKGRIIINADTSKNQLSLQLKSVTPEDAAVYYCARS-SDMIFGII-VHLDYWGQGSLVTVSS
SRR2899819.2345	QVQLQQSGPGLVKPSQTLSLTCAVSGDSVSSNSVAWNWIRQSPSRGLEWLGRTYYRSKWYNDYAVSVKGRISINPDTSYNQLSLQLTSVTPEDAAVYYCARS-SDIVFGVV-AHLDYWGQGTLVTVSS
SRR2899731.4452	QVQLQQSGPGLVKPSQTLSLTCAISGDSVSSNSAAWNWIRQSPSRGLEWLGRTYYRSKWYNDYAVSVKSRITINPDTSKNQFSLQLNSVTPEDTAVYYCARD-GIRIFGLLIGVFDYWGQGALVTVSS
SRR2899731.3725	QIQLQQSGPGLVKPSQTLSLTCAISGDSVSSNSAAWNWIRQSPSRGLEWLGRTYYRSKWYSDYAVSVKSRITINPDTSKNQFSLQLNSVTPEDTAVYYCARD-GIRIFGLLIGVFDYWGQGALVTVSS
SRR2899731.19929	QVQLQQSGPGLVKPSQTLSLTCAISGDSVSSNSAAWSWIRQSPSRGLEWLGRTYYRSKWYYDYAPSVKSRMTFNPDTSKNQFTLQLDSVSPEDTAVYYCART-GITIFGVLIPALDVWGQGTTVTVSS
SRR2899882.400	QVQLQQSGPGLVKPSETLSLTCAISGDSVSSNSAAWNWIRQSPSRGLEWLGRTYYRSKWYNDYADSVRSRITINPDTSKNQFTLQLKSVTPEDTAVYYCARE-SITIFGVIINAFDIWGQGTMVTVSS
SRR2899882.11444	QVQLQQSGPGLVKPSETLSLTCAIAGESVSSNSAAWNWIRQSPSRGLEWLGRTYYRSKWYNDYADSVGSRITINPDTSKNQFTLQLKSVTPEDTAIYYCARE-SITIFGLIINAFDIWGPRGQWSPSL
SRR2899884.46167	QIQLQQSGPGLVKPSQTLSLTCSISGDTVTNNYAAWDWIRQSPTRGLEWLGRTFYRSKWYKEYALSVKSRLTISPDTSKNQISLQLSSVTPEDTAVYYCARA-GITIFGLITGGLDYWGQGSLVTVSS
SRR2899884.39218	QIQLQQSGPGLVKPSQTLSLTCSISGDTVTNNYAAWDWIRQSPTRGLEWLGRTFYRSKWYKEYALSVKSRLTISPDTSKNQISLQLNSVTPEDTAMYYCARA-GITIFGLITGGLDYWGQGSLVTVSS
SRR2899884.40156	QVQLQQTGPGLVKPSQTLSLTCAISGDSVASNSAAWNWIRQTPSKGLEWLGRTYYRSKWYNDYAVSVKSRVTINSDTSKNQFSLQLNSVTPEDTAVYYCARA-GITIFGVVIGGLDYWGQGILVTVSS
SRR2899884.41756	QVQLQQSGPGLVKPSQTLSLTCAVSGDSVTSTSAAWDWIRQSPSRGLEWLGRTYYRSKWYTDYAVSVKSRVMINSDTSKNQFSLQLNSVTPEDTAVYYCARA-GITVFGLVTGGLDYWGQGTLVTVSS
SRR2899831.7409	QVQLQQSGPGLVKPSQTLSLTCAISGDSVSSNSAAWNWIRQSPSRGLEWLGRTYYRSKWFNDYAVSVKSRISIKPDTSKNQFSLQLNSVTPEDTAVYYCARV-GITIFGLVIFAFDVWGQGTMVTVSS
SRR2899881.71253	QVQLQQSGPGLVKPSQTLSLTCAISGDSVSSNSAAWNWIRQSPSRGLEWLGRIYYRSQWYDDYAPSVKGRMTINPDTSKNQFSLQLKSVSLEDTAIYYCARA-RLTIFGLLIPAFEDWGQGTLVTVSS
SRR2899882.9127	QVQLQQSGPGLVKPSQTLSLTCAISGDSVSSNGASWNWIRQSPSRGLEWLGRTYFRSKWYYDYAVSVKSRISFNPDTSKNQFTLHLKNVTPEDTAVYYCARV-GITIFGILIEAFDYWGQGTLVTVSS
SRR2899884.28946	QIQLQQSGPGLVKPSQTLSLTCAISGDSVSSSTVAWNWIRQSPSRGLEWLGRTYYRSKWYSNYSPSVKSRITITPDTPKNQFSLQLNSVTPDDSAVYYCARA-GITIFGVLVGGLDYWGQGTVVTVSS
