# Supplementary material for: Sequence-Signature Optimization Enables Improved Identification of Human HV6-1-Derived Class Antibodies That Neutralize Diverse Influenza A Viruses
Source: Front Immunol. 2021 May 31;12:662909. doi: 10.3389/fimmu.2021.662909 (PMC8201785; doi:10.3389/fimmu.2021.662909)
Supplement: Supplementary file 6 [file DataSheet_6.pdf]

Dataset S6A. Nine heavy chain sequences identified from sequence signature search using HV6-1 class signature version 1. Missing residues were filled in with germline amino acids.

```
204    QVQLQQSGPGLVKPSQTLSLTCAISGDSVSSNSAAWNWIRQSPSRGLEWLGRTYYRSKWYNDYAVSVKSRITINPDTSKNQFSLQLNSVTPEDTAVYYCARDP-ITIFGVGRGAFDIWGQGTMVTVSS
321    QVQLQQSGPGLVKPSQTLSLTCAISGDSVSSNSAAWNWIRQSPSRGLQWLGRTYYRSKWYNDYAVSVKSRISIFPDTSKNQVSLQLNSVTPEDTAVYYCTRA-SGLVFGVVIS-LDVWGKGTTVTVSS
1431   QVQLQQSGPGLVKPSQTLSLTCAISGDSVSSNSAAWNWIRQSPSRGLEWLGRTYYRSKWYNDYAVSVKSRITINPDTSKNQFSLQLNSVTPEDTAVYYCARDHSITIFGVPQYGMDVWGQGTTVTVSS
661    QVQLQQSGPGLVKPSQTLSLTCAISGDSVSSNSAAWNWIRQSPSRGLEWLGRTYYRSKWYNDYAVSVKSRITINPDTSKNQFSLQLNSVTPEDTAVYYCARGGDVTIFGVVTDYFDYWGQGTLVTVSS
1093   QVQLQQSGPGLVKPSQTLSLTCAISGDSVSSNSAAWNWIRQSPSRGLEWLGRTYYRSKWYNDYAVSVKSRITINPDTSKNQFSLQLNSVTPEDTAVYYCARGR-ITIFGVVAYSFDYWGQGTLVTVSS
117    QVQLQQSGPGLVKPSETLSLTCAISGDSVSSNSAAWNWIRQSPSRGLEWLGRTYYRSKWYNDYAVSVKSRITINPDTSKNQFSLQLNSVTPEDTAVYYCAREAEITIFGVVIDAFDIWGQGTMVTVSS
357    QVQLQQSGPGLVKPSQTLSLTCAISGDSVSSNSAAWNWIRQSPSRGLEWLGRTYYRSKWYNDYAVSVKSRITINPDTSKNQFSLQLNSVTPEDTAVYYCARDPLITIFGVGPYGMDVWGQGTTVTVSS
760    QVQLQQSGPGLVKPSQTLSLTCAISGDSVSSNSAAWNWIRQSPSRGLEWLGRTYYRSKWYNDYAVSVKSRVTINPDTSKNQLSLQLNSVTPEDTAVYYCVRS-SPMVFGVVH-AFDIWGQGTLVTVSS
895    QVQLQQSGPGLVKPSQTLSLTCAISGDSVSSNSAAWNWIRQSPSRGLEWLGRTYYRSKWYNDYAVSVKSRITINPDTSKNQFSLQLNSVTPEDTAVYYCAREG-VTIFGVVT-SFDYWGQGTLVTVSS
```

Dataset S6B. Alignment of partial light chain sequences identified from paired heavy:light chain sequencing. Important contact residues were colored red.

```
Germline gene and known HV6-1 class (and associated light chain gene)

IGKV1-39                  DIQMTQSPSSLSASVGDRVTITCRASQSI--SSYLNWYQQKPGKAPKLLIYAASSLQSGVPSRFSGSGSGTDFTLTISSLQPEDFATYYCQQSYSTPP
MEDI8852 (IGKV1-39)       DIQMTQSPSSLSASVGDRVTITCRTSQSL--SSYTHWYQQKPGKAPKLLIYAASSRGSGVPSRFSGSGSGTDFTLTISSLQPEDFATYYCQQSR------TFGQGTKVEIK

IGKV3-20                  EIVLTQSPGTLSLSPGERATLSCRASQSV-SSSYLAWYQQKPGQAPRLLIYGASSRATGIPDRFSGSGSGTDFTLTISRLEPEDFAVYYCQQYGSSPP
54.f.01 (IGKV3-20)        EIVLTQSPGTLSLSPGERATLSCRASQSV-SSSYLAWYQQKPGQSPRLLIYGTSTRATGIPDRFSGSGSGTEFTLTITRLEPEDFAVYYCQQFDGSH--FTFGPGTKVDIK
56.a.09 (IGKV3-20)        EIVLTQSPGTLSLSPGERATLSCRASQSV-ASSYLAWYQQKPGQAPRLLIYGASSRATGVPDRFSGSGSGTDFILTISRLEPEDFAVYYCQQYDGSQ--YTFGQGTKLEIK
58-6E11 (IGKV3-20)        EIVLTQSPGTLSLSPGERATLSCRASQNV-AVSYLAWYQQKPGQAPRLLIYGASSRATGIPDRFSGSGSGTDFTLTISRLEPEDFAVYYCQQYATSPRALTFGGGTKVEIK
58-6B09 (IGKV3-20)        EIVLTQSPGTLSLSPGERATLSCRASQSV-SSSYLAWYQQKPGQAPRLLIYGASSRATGIPDRFSGSGSGTDFTLTISRLEPEDFAVYYCQQYATSPRALTFGGGTKVEIK
58-6E04 (IGKV3-20)        EIVLTQSPGTLSLSPGERATLSCRASQSV-SSSYLAWYQQKPGQAPRLLIYGTSSRATGIPDRFSGSGSGTDFTLTISRLEPEDFAVYYCQQYATSPRALTFGGGTKVEIK
58-6F03 (IGKV3-20)        EIVLTQSPGTLSLSPGERATLSCRASQSV-ASSYLAWYQQKPGQAPRLLIYGTSSRATGIPDRFSGSGSGTDFTLTISRLEPEDFAVYYCQQYATSPRALTFGGGTKVEIK
54-1G05 (IGKV3-20)        EIVLTQSPGTLSLSPGERVTLSCRASQTV-YNSYLAWYQQKPGQAPTLLIYGTSTRATGVPDRFSGSGSGTVFTLTISRLEPEDFAVYFCQQYSTSPRALTFGGGTKVEIK

IGKV1-5                   DIQMTQSPSTLSASVGDRVTITCRASQSI--SSWLAWYQQKPGKAPKLLIYDASSLESGVPSRFSGSGSGTEFTLTISSLQPDDFATYYCQQYNSYSP
IGKV1-33                  DIQMTQSPSSLSASVGDRVTITCQASQDI--SNYLNWYQQKPGKAPKLLIYDASNLETGVPSRFSGSGSGTDFTFTISSLQPEDIATYYCQQYDNLPP
IGKV1-NL1                 DIQMTQSPSSLSASVGDRVTITCRASQGI--SNSLAWYQQKPGKAPKLLLYAASRLESGVPSRFSGSGSGTDYTLTISSLQPEDFATYYCQQYYSTPP
IGLV1-40                  QSVLTQPPSVSGAPGQRVTISCTGSSSNIGAGYDVHWYQQLPGTAPKLLIYGNSNRPSGVPDRFSGSKSGTSASLAITGLQAEDEADYYCQSYDSSLSGS
IGLV1-51                  QSVLTQPPSVSAAPGQKVTISCSGSSSNI-GNNYVSWYQQLPGTAPKLLIYDNNKRPSGIPDRFSGSKSGTSATLGITGLQTGDEADYYCGTWDSSLSAG
IGLV2-14                  QSALTQPASVSGSPGQSITISCTGTSSDVGGYNYVSWYQQHPGKAPKLMIYEVSNRPSGVSNRFSGSKSGNTASLTISGLQAEDEADYYCSSYTSSSTL
IGLV3-1                   SYELTQPPSVSVSPGQTASITCSGDKL---GDKYACWYQQKPGQSPVLVIYQDSKRPSGIPERFSGSNSGNTATLTISGTQAMDEADYYCQAWDSSTA

Signature identified HV6-1 antibodies with associated light chain gene
PRJNA260556:
SRR1585249.362669_KV1-5     -------------------------------------QQKPGKAPKLLIYRASSFQSGVPSRFSGSGSETEFTLTISRLQPDDFATYYCQQYHSY--WETFGQGTKVEIK
SRR1585249.4432058_LV1-51*  ----------------------------------------------LLLYDNNKRPAGIPDRFSCSKSGTSAPLGITGLQTGDEADYFCGTWDNSLLAVLFGGGTWLTVL
SRR1585249.183903_LV2-14    ----------------------------------------------LMIYEGSKRPSGVSHRFSGSKSSNTASLTISGLHAEDEADYFCSSYSSGSPPYVFGAGTKVTVL
SRR1585265.157719_KV1-39*   -------------------------------------QQKPGTAHKFLLYPAYSMQSGVPSRFRGSGSGTDFTVTISSLQPEDFATYYCQHSYSI--PFTFGPGNKVDIK
SRR1585265.2822657_KV1-39   -------------------------------------QQKPGKAPNFLIYAASSLESGVPSRFSGSGSGTDFTLTISSLQPEDFATYYCQQSYSV--PFTFGPGTKVDIK

PRJNA315079:
SRR3273596.3285188_KV1-33*  -------------------TITCQASQDI-SNYLNWYQQKPGKAPKLLIYDAPNLETGVPSRFSGSGSGTDFTFTISSLQPEDIATYYCQQYDNL--PRSFGQGTKLEIK
SRR3273596.5223220_KV1-33   --------------------ITCTASQDI-SSYLNWYQQKPGKAPKLLIYDASNLETGVPSRFSGSGSGTDFTFTISSLQPEDIATYYCQQYDNL-EGDRFGGGTKVEIK
SRR3273593.3663150_KV1-39*  --------------------------------------QKPGKAPKLLIYAASSLQSGVPSRFSGSGSGTDFTHTISRLQPEDFATYYCQQSYST-PLVTFGQGTRLEIK
SRR3273595.2555017_LV1-40*  ----------------------------------------------LLINGNVNRPSGVPDRFAGSKSGATTSLTITGRQAEDEADYYCQSYDISLSGYVFGTGTKFTVL
SRR3273595.3761138_LV1-40*  ----------------------------------------------LLSYGNSNRPSGVPDRFSGSKSGTSASLAITGLQAEDEADYYCQSYDSSLSGYVFGTGAKVTVL
SRR3273595.490959_LV1-40*   ----------------------------------------------LLIYGNSNRPSGVPDRFSGSKSSTSASLAITGLQAEDEADYYCQSYDSSLSGYVFGTGTKVTVL
SRR3273595.609148_LV1-40*   ----------------------------------------------LLIYGNSNRPSGVPDRFAGSKSGTSASLAITGLQAEDEADYYCQSYDSSLSGYVFGTGTKVTVL

PRJNA523171:
SRR8592593.597334_LV1-40    ----------------------------AGYDVHLY-QQLPGTAPKLLIYGNNNRPSGVPDRFSGSKSGTSASLAITGLQAEDEADYYCQSYDSSLSGVVFGGGTRLTVL
SRR8592593.1353914_LV1-51*  ----------------------------GNNPVLCY-QQYPATAPTRLIYKVNERPSGIPERFSGSKSGTSATLDISGLQTGDEADYYCATWDISLNSGVFGGGTKLTVL
SRR8592593.936_LV2-14       ----------------------------GASTYAAWSQLHPAKAPILLTLDVPTRTSGVSTRLSGSKSGNTASLTISGLQADDAADYYCSSYSIRGTY-VFGTGTKVTVL
SRR8592599.704723_LV3-1     -------------------------------KFTCWCNQKPGQSPLLVIYQNRERPSGIPERFSGSNSGNTATLTISETQVFDEADYFCQVWDSHTASFVFGTGTQVTVL
SRR8592599.552669_KV1-39    ---------------------RCRASQGA-KRYLNWSQQKRAQDPQFLISGASELQREVPPSCSGNGSGTEFRRTVSRLQPEDFADYYCQQRCGNHP--TFGRGTQVEIK
SRR8592593.215710_KV1-NL1   -------------------TITCRARQGI-YNSLAWYQQKPGEAPKLLLYDASKLESGVPARFSGSGSGTDYTLSISSLQPEDFATYYCHQYYNT-PW-TFGQGTKVEIK
SRR8592593.67523_KV3-20     ------------------ATLSCRASQSVSSSYFAWYQQKPGQAPRLLIYGASSRATGIPDRFSGSGSGTDFALTISRLEPEDFGVYYCQEYH------TFGQGTKVEIK
SRR8592593.964178_KV3-20    ---------------GARATLSCRAGQSLSSSYVAWYQQKPGQAPRLLIYGASSRATGIPDRFSGSGSGTDFLLTISRLEPEDSAVYFCQHYH------TFGQGTKWKSN
SRR8592595.390409_KV3-20    -----------------RDNAAGRAGRRLGGGYVAWYEQKPGQAPGVGSCGASGRATGIPDRLSGSGSGTDFRRSDSRLEPEDCAVYYCKHYH------TFGQGTKVEIK
SRR8592595.1043224_KV3-20   ----------------------CRASTGSMRSYQVWYKQTPGQAPRLRVYRAASRPTGIPDRCGGRGSGTDFTLTISRLDPGEFAGYCCRDYL------TFGQGTKVEIQ
```

* Light chain selected by version 1 signature but not selected by version 2 signature.
