# Supplementary material for: Sequence-Signature Optimization Enables Improved Identification of Human HV6-1-Derived Class Antibodies That Neutralize Diverse Influenza A Viruses
Source: Front Immunol. 2021 May 31;12:662909. doi: 10.3389/fimmu.2021.662909 (PMC8201785; doi:10.3389/fimmu.2021.662909)
Supplement: Supplementary file 8 [file Table_1.pdf]

Table S1A. Accession code, number of reads, and number of HV6-1 class influenza antibodies for each of the influenza vaccine studies analyzed.

			Version 0 signature (Joyce et al. 2006 Cell)		Version 1 signature		Version 2 signature	
Accession Code	Heavy Chain Reads	Total HV6-1 reads	# of reads (# cluster)	# functional sequence cluster representative (fraction)	# reads (# cluster)	# functional sequence cluster representative (fraction)	# reads (# cluster)	# functional sequence cluster representative (fraction)
PRJNA176314	4,152,130	308,593	14 (1)	1	210 (42)	11 (0.26)	83 (22)	9 (0.41)
PRJNA301150	1,731,855	24,658	1	1	283 (17)	14 (0.82)	278 (11)	10 (0.91)
PRJNA324093	15,456,937	142,973	0	-	5 (3)	-	2(2)	-
Sum of above	21,340,922	476,224	2	2 (1.0)	498 (62)	25 (0.40)	363 (35)	19(0.54)
Phs000666	1,731,771	24,658	0	-	16 (9)	5 (0.56)	16(9)	5 (0.56)

Table S1B. Number of reads, and number of HV6-1 class influenza antibodies from NGS samples of healthy donors.

	Healthy Donor	Total IgM Reads	Total HV6-1 reads	Version 0 signature present	Version 1 signature present (# cluster)	Version 2 signature present (# cluster)
PRJNA511481	HIP1	126,000,385	152,686	0	53 (11)	50 (9)
	HIP2	158,266,858	281,979	0	326 (36)	217 (20)
	HIP3	125,235,487	331,429	0	19 (4)	0
PRJNA406949	316188	7,906,619	35,151	0	15(4)	14(3)
	326650	23,249,481	270,973	0	155(79)	115(58)
	326651	59,287,210	634,779	0	353(171)	215(84)
	326713	78,227,028	764,271	0	401(215)	269(133)
	326737	17,593,490	341	0	1	1
	326780	38,877,576	581,378	0	741(40)	51(13)
	326797	30,199,839	625,436	0	192(82)	104(52)
	326907	12,579,986	100,537	0	26(11)	25(10)
	327059	25,388,032	314,259	0	96(36)	73(26)
	D103	11,197,546	106,493	0	36(11)	25(9)

Table S1C. Number of reads, and number of HV6-1 class influenza antibodies from NGS samples of naïve B cells and cord blood.

	Donor	Total Reads	Total HV6-1 reads	Version 0 signature present	Version 1 signature present (# cluster)	Version 2 signature present (# cluster)
PRJNA511481	CORD1(4)	3,338,669	187,094	0	54(6)	49(3)
	CORD2(2)	4,434,204	200,622	0	215(10)	83(3)
	CORD3(3)	3,215,474	89,741	0	55(8)	22(3)
PRJNA315079	Donor1	4,984,508	87,335	0	1	0
	Donor2	1,433,424	22,429	0	15(4)	0
	Donor3	1,832,971	17,238	0	2(2)	1

Table S2. Cryo-EM Data Collection and Refinement Statistics.

	SRR2899884.46167H Fab in complex with influenza A/Victoria/361/2011 HA
**EMDB ID**	EMD-22804
**PDB ID**	7KC1
Data Collection	
Microscope	FEI Titan Krios
Voltage (kV)	300
Electron dose (e^-/Å^2)	71.06
Detector	Gatan K2 Summit
Pixel Size (Å)	1.07
Defocus Range (µm)	-0.3 to -4.1
Magnification	22500
Reconstruction	
Software	cryoSparcV2.12
Particles	29,735
Symmetry	C3
Box size (pix)	352
Resolution (Å) ($FSC_{0.143}$)	3.4
Refinement	
Software	Phenix 1.18
Protein residues	2106
Chimera CC	0.84
EMRinger Score	2.61
R.m.s. deviations	
Bond lengths (Å)	0.003
Bond angles (°)	0.555
Validation	
Molprobity score	1.51
Clash score	5.09
Favored rotamers (%)	100
Ramachandran	
Favored regions (%)	96.4
Disallowed regions (%)	0.0

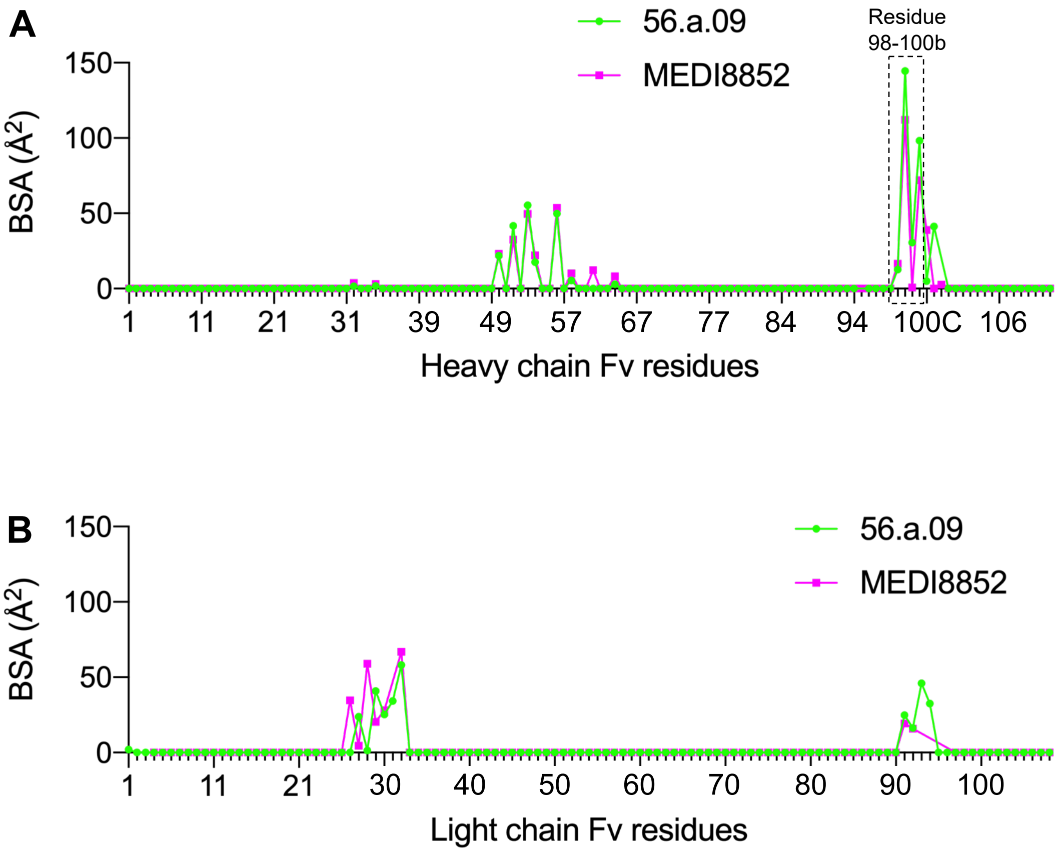

Figure S1. Buried surface area (BSA) of HV6-1 class influenza antibodies 56.a.09 and MEDI8852 in complex with hemagglutinin. (A) heavy chain Fv residues (B) Light chain Fv residues.

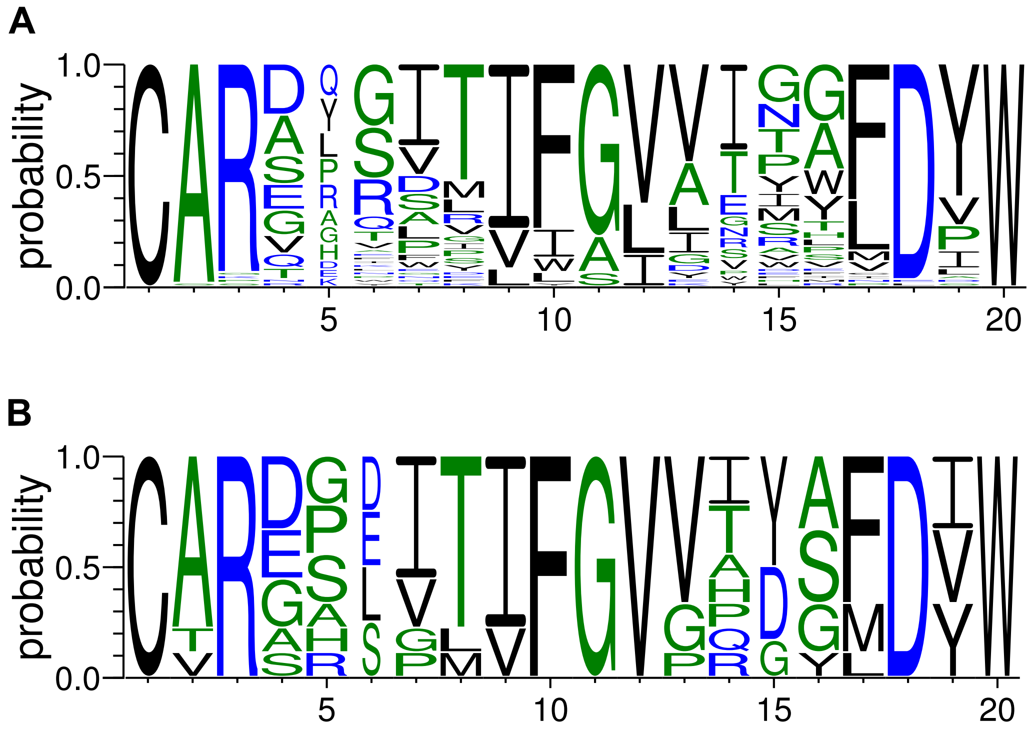

Figure S2. Sequence Logo representation of the CDR H3 from (A) 62 sequences identified from NGS datasets PRJNA176314, PRJNA301150, and PRJNA324093 using HV6-1 class signature version1, and (B) 9 sequences identified from NGS dataset Phs000666 using HV6-1 class signature version 2.

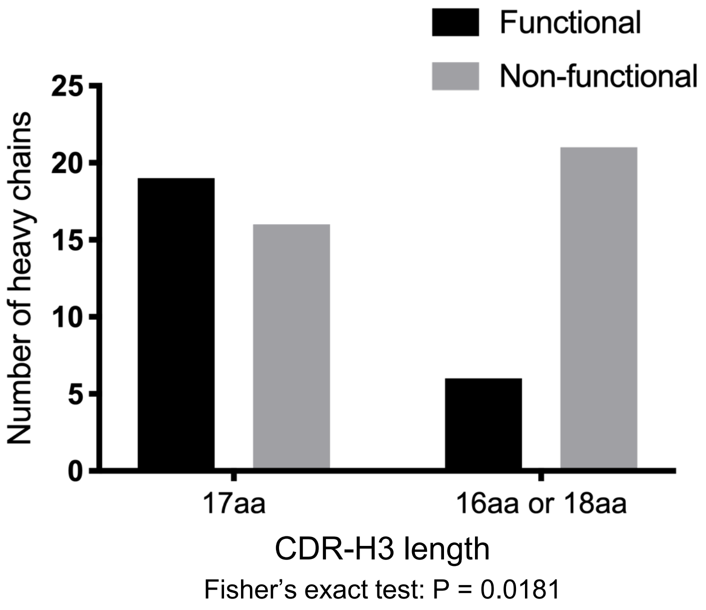

Figure S3. 2x2 contingency analysis of the functionality of signature identified heavy chain sequence versus CDR-H3 length. P-values were calculated using two-tailed Fisher's Exact test.

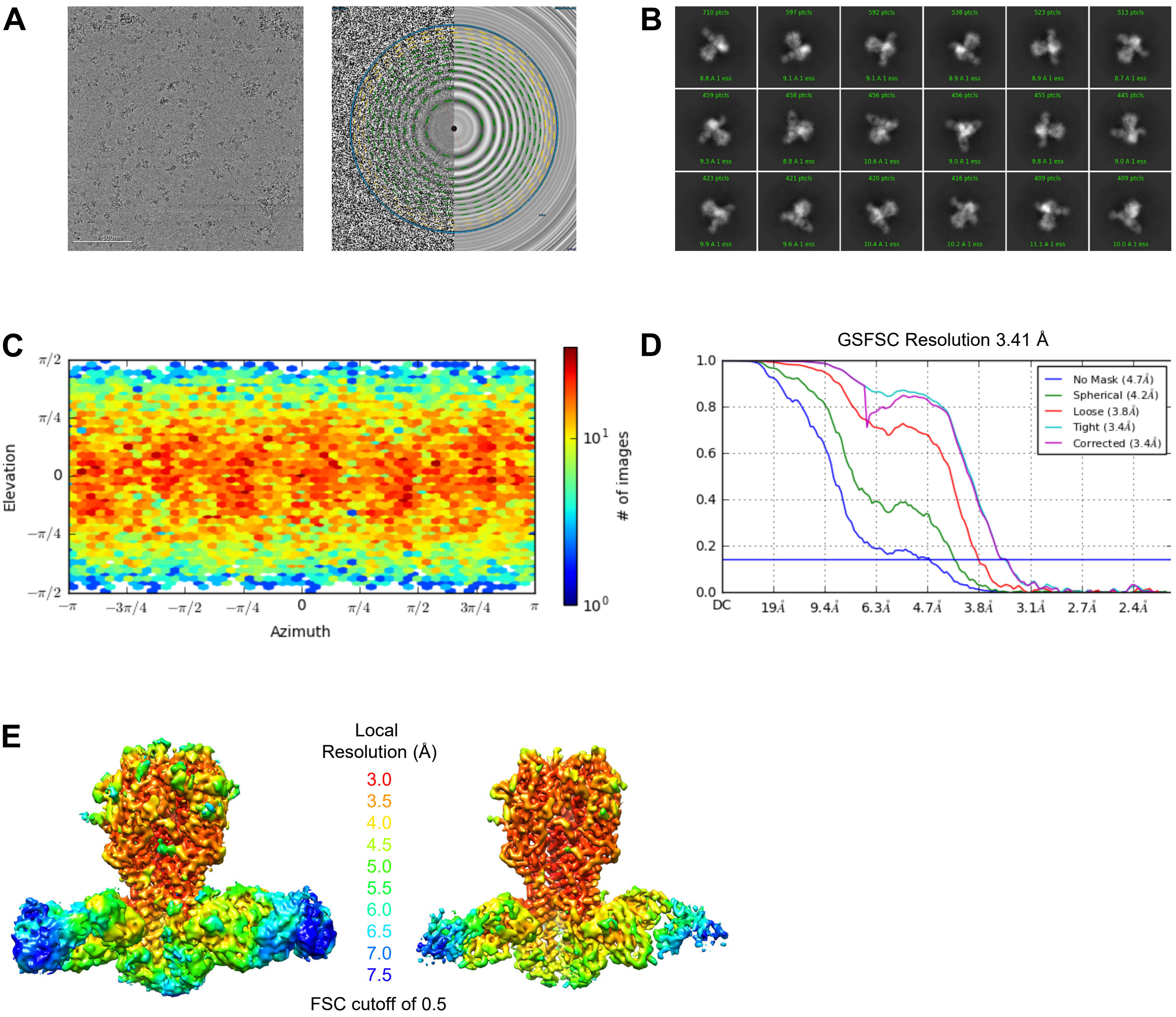

Figure S4. Cryo-EM Details of SRR2899884.46167H+MEDI8852L in complex with H3N2 Victoria HA. (A) Representative micrograph and CTF of the micrograph are shown. (B) Representative 2D class averages are shown. (C) The orientations of all particles used in the final refinement are shown as a heatmap. (D) The gold-standard Fourier shell correlation resulted in a resolution of 3.41 Å using non-uniform refinement with C3 symmetry. (E) The local resolution of the full map is shown generated through cryoSPARC using an FSC cutoff of 0.5. Two contour levels are shown.

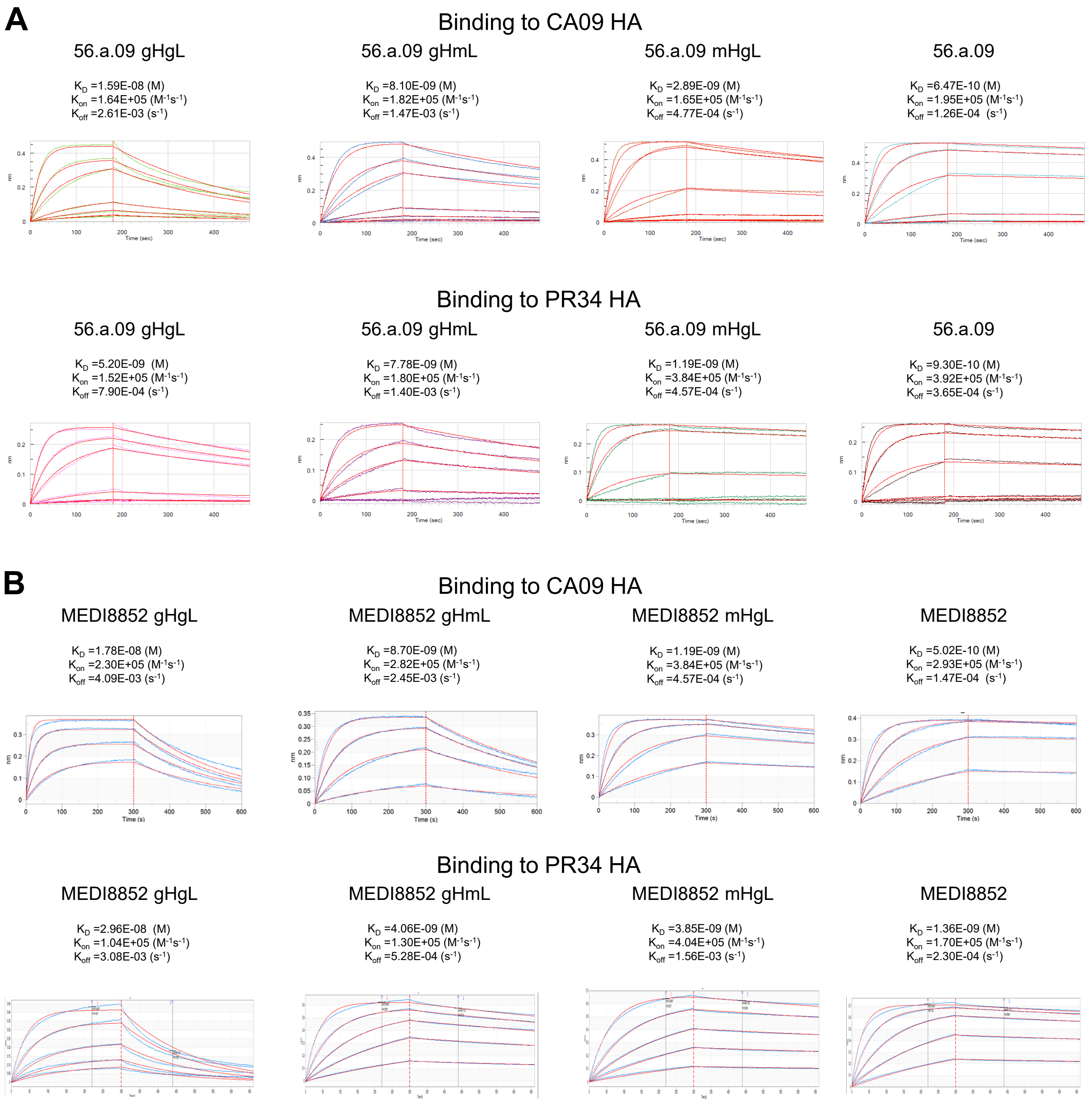

Figure S5. Binding of wild type, mHgL (light chain reverted to germline), gHmL (heavy chain reverted to germline) and gHgL (both chain reverted to germline) to CA09 and PR34 HA, respectively, for (A) 56.a.09, and (B) MEDI8852, using Bio-Layer Interferometry.
